# Regional variation in the incidence rate and sex ratio of multiple sclerosis in Scotland 2010–2017: findings from the Scottish Multiple Sclerosis Register

**DOI:** 10.1007/s00415-019-09413-x

**Published:** 2019-06-11

**Authors:** Patrick K. A. Kearns, Martin Paton, Martin O’Neill, Chrissie Waters, Shuna Colville, James McDonald, Ian J. B. Young, Dan Pugh, Jonathon O’Riordan, Belinda Weller, Niall MacDougall, Tom Clemens, Chris Dibben, James F. Wilson, Marcia C. Castro, Alberto Ascherio, Siddharthan Chandran, Peter Connick

**Affiliations:** 1 Anne Rowling Regenerative Neurology Clinic, University of Edinburgh, Chancellor’s Building, 49 Little France Crescent, Edinburgh, EH16 4SB UK; 2 Centre for Clinical Brain Sciences, University of Edinburgh, Edinburgh, UK; 3 Information Services Division, NHS Scotland, Edinburgh, UK; 4 Department of Neurology, NHS Grampian, Aberdeen, Scotland UK; 5 Edinburgh Royal Infirmary, NHS Lothian, Edinburgh, Scotland UK; 6 Department of Neurology, NHS Tayside, Dundee, Scotland UK; 7 Department of Neurology, NHS Lanarkshire, Glasgow, Scotland UK; 8 Department of Human Geography, University of Edinburgh, Edinburgh, Scotland UK; 9 Centre for Global Health Research, Usher Institute for Population Health Sciences and Informatics, University of Edinburgh, Edinburgh, UK; 10 MRC Human Genetics Unit, Institute of Genetics and Molecular Medicine, University of Edinburgh, Edinburgh, Scotland UK; 11 Department of Global Health and Populations, Harvard T.H. Chan School of Public Health, Boston, MA USA; 12 Department of Epidemiology, Harvard T.H. Chan School of Public Health, Boston, MA USA

**Keywords:** Epidemiologic methods, Central nervous system diseases, Registry, Autoimmune diseases, Epidemiology

## Abstract

**Background:**

Fifteen regional studies published over the last six decades surveying prevalence, mortality and hospital admissions have suggested that Scotland is amongst the highest risk nations for multiple sclerosis (MS) in the world. However, substantial intranational variation in rates (between regions) has been described in numerous countries, including in the only previous Scottish national survey, which used hospital admission data, to address this issue. Against this backdrop, the Scottish Multiple Sclerosis Register (SMSR) was established in 2010 to prospectively collect nationally comprehensive incidence data and to allow for regional comparisons.

**Methods:**

Here, we present the SMSR and analyse the variation in crude and age–sex standardized incidence rates, lifetime risk (cumulative incidence), and the sex distribution of cases and rates, between the 14 administrative Health Boards or regions of Scotland: 01 January 2010 to 31 December 2017.

**Results:**

The overall incidence rate for Scotland was 8.76/100,000 person-years (standardized: 8.54). Regional incidence rates varied significantly—up to threefold—between Health Boards (*p* < 1 × 10^–13^). The national female-to-male sex ratio was 2.3:1, but this too varied regionally (outlier regions result in a range from 1.0 to 4.2:1). Lifetime risk ranged from 19.9/1000 for females in Orkney (58.98°N) to 1.6/1000 for males in the Borders (55.60°N). Comparison with a previous national survey suggests that these differences are longstanding. In 6 of 14 regions the lifetime risk for women exceeds 1%.

**Conclusions:**

This study introduces a national incidence register: a valuable research tool and the result of substantial public investment. The wide variation in incidence rates and sex ratios between regions, in a relatively homogenous population, raises questions for future study.

**Electronic supplementary material:**

The online version of this article (10.1007/s00415-019-09413-x) contains supplementary material, which is available to authorized users.

## Introduction

Broad consensus exists for genetic susceptibility interacting with potent environmental risk factors in the pathogenesis of multiple sclerosis (MS) [1–3]. Nevertheless, a detailed account of the causal chain remains elusive. One aspect of MS epidemiology that remains unexplained is the well recognized within-country regional variation in MS rates [4–7]. In many countries, differences in regional rates have been wide (up to tenfold) and persistent in the same regions across period cohorts, i.e. relative differences in regional rates persist despite different generations of patients being diagnosed by different generations of neurologists [5]. There may be an opportunity to identify novel contributors to aetiology by studying these variations, because the list of possible causes (genetic or environmental) is more limited between relatively similar sub-national regional populations than between disparate international ones. Addressing this, however, will require high quality and geographically precise epidemiological datasets that can guide targeted genetic, seroepidemiological, and biome research.

Scotland represents a powerful environment to study MS due its very high incidence of the disease. This is reflected in a strong history of Scottish MS epidemiological study over several decades. Fifteen surveys have been published since the first by Sutherland in 1956 [8], which have consistently shown a pattern of increasing prevalence and high rates by international standards [9, 10]. In addition, there are technical advantages particularly for making between-region comparisons in Scotland. These include a stable and homogeneous population of 5.3M, a unitary and free health-care provider system (NHS) and e-health infrastructural strengths such as a nationwide patient identifier (CHI) assigned at birth. The CHI enables lifelong tracking for all contact with public services including vaccinations, hospital admissions, prescriptions, etc., and consequently the creation and alignment of detailed datasets of social and health care interactions. However, only one study has attempted to document within-Scotland variation in rates using a national sample covering a single period [11].

In 2010, recognizing MS as an important public health problem, the Scottish Government established the Scottish MS Register (SMSR), a prospective incidence register, mandating the recording of all newly diagnosed cases of MS. The primary purpose was to ensure that all persons diagnosed with MS were offered contact with an MS specialist nurse within ten working days of diagnosis and, secondarily, to routinely and systematically collect comprehensive data for epidemiological research [12]. Our aims in this paper are twofold: first to present the SMSR to highlight its potential as a research tool, and second to analyse the first 8 years of incidence data captured therein to study the regional variability in MS rates between the 14 “Health Board” regions of Scotland.

## Methods

### Data

The SMSR aims for comprehensive ascertainment by coupling routine data collection with a continuous audit of service quality. The ten working day requirement is an “essential criteria” of a national standard [Standard 15.2 of the Clinical Standards for Neurology Services (2009)] which requires Health Boards to meet this target [13]. MS specialist nurses are obliged to report data on this outcome accurately and promptly after diagnosis which is then confirmed by the treating neurologist. Completeness and accuracy are continuously monitored by Information Services Division (ISD) of National Health Service (NHS) National Services Scotland (NSS).

Individual level SMSR data, including age, date and address at the time of diagnosis, were extracted in an anonymized form. Census data, publically available for Health Board populations, were used for the denominators for all calculations based on the Scottish national census 2011 (https://www.scotlandscensus.gov.uk/) [14].

### Inclusion and exclusion criteria

Adults (legally persons ≥ 16 years old), newly diagnosed with MS in Scotland after 01/01/2010, as defined by the Revised McDonald Criteria 2005 [15], are routinely recorded on the SMSR. We included all those persons with MS (pwMS) on the register diagnosed up to 31 December 2017. Persons with possible MS or clinically isolated syndrome are excluded (https://www.msr.scot.nhs.uk/).

### Health Board geography

Scotland has a universal health care system, publically owned and operated, free at the point of use (NHS). The great majority of health care in Scotland is delivered by the NHS. Private neurologists typically also work in the NHS (and refer to MS services via specialist nurses) and probably all pwMS in Scotland, who have been diagnosed, use some NHS services. NHS Scotland is separated into 14 territorial Health Boards which were the aggregate unit of geography used in this study. Health Board latitude was defined as the latitude of the administrative centre.

### Ethics

Formal research ethics approval was not required as audit data were de-identified, but peer-review by the SMSR steering committee and independent internal (ISD) review by an NHS employed consultant of public health ensured the project was in line with the aims and objectives of the SMSR and consistent with usual public health practice for the use of routinely collected data obtained without explicit consent.

### Statistical analyses

To allow for external comparisons unconfounded by population age–sex structure, direct-standardization of incidence rates was performed to the 2013 European Standard Population (ESP) (https://www.ons.gov.uk). Indirect standardization of regional rates was conducted using national rates as the referent for between-health-board comparisons. Maps were created using QGIS v2.18.23 using publicly available shapefiles (data.gov.uk). The cartogram was created using GeoDa v1.12.

Lifetime (risk) cumulative incidence rates (CIR) were calculated for each region as:

$${\text{CIR}} = 1 - {\text{exp}}\left[ { - \mathop \sum \limits_{{i = 1}}^{n} ({\text{ID}}_{i} )w_{i} } \right],$$

where *i* = age stratum, ID = incidence density for the *i*th age stratum, and *w*_*i*_ = the width in years of the *i*th age stratum. This measure has the advantage of incorporating (in ID strata) the population size of the strata and so is comparable across populations of different age-structures. CIRs are the proportion of population affected over a given period, here calculated to reflect lifetime risk and are presented per 1000 persons.

Statistical significance was defined using an *α* of 0.05 throughout. Omnibus test of statistical significance of variation in rates by region was tested using a 14-sample Chi-squared test. Associations were inspected visually and where appropriate linear relationships were analysed using ordinary and weighted least squares regression models. Pearson’s correlation coefficients were generated for bivariate analyses. Simulated rates under a null hypothesis of complete spatial randomness were calculated by randomly allocating each of *n* = 3680 cases to Health Boards proportional to population size 100,000 times in order to create empirical distributions under the null. *p* values for observed rates occurring under the null were calculated by comparing observed rates to the empirically derived null distributions and correcting for *m* = 14 independent tests using the Bonferroni method (*α*/*m*). Statistical analyses, simulations and tests were conducted using R and R Studio (v. 3.5.1).

The dataset used here is available subject to approval of the SMSR steering committee and NHS NSS ISD.

## Results

### The Scottish MS Register

**Fig. 1:**
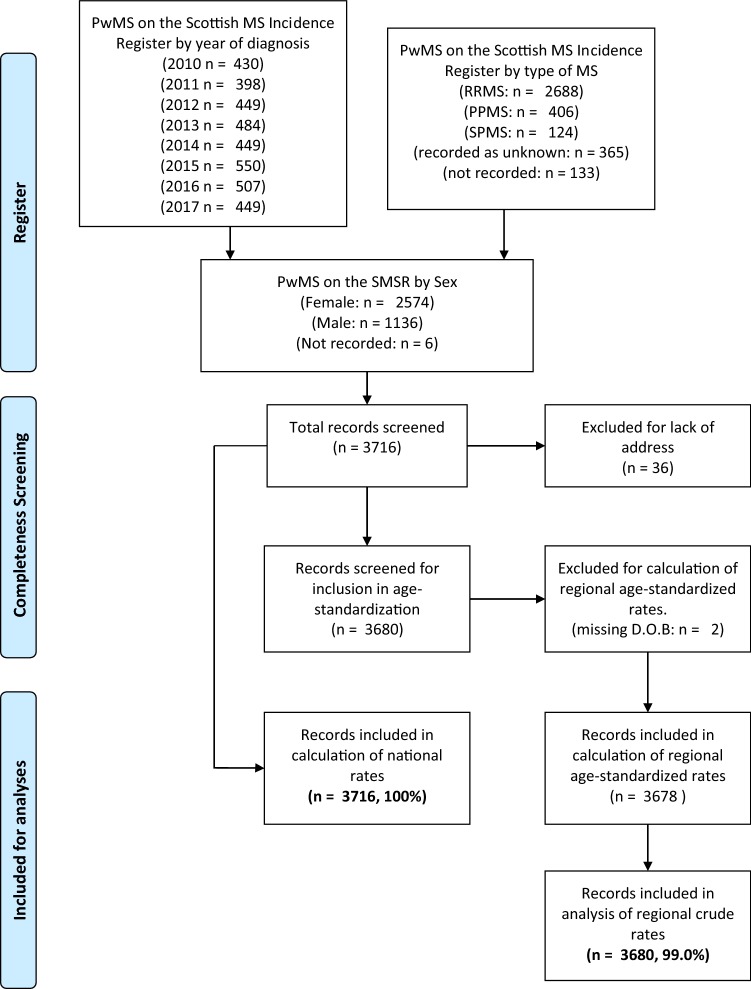
Flow diagram of included data

3680/3716 (99.0%) cases were included in the regional analyses with 36 persons excluded due to incomplete address data (Fig. 1).

The resident populations within Health Boards are presented in Table 1, showing regional variation in population number and density. The majority of Scotland’s population lives in the “central belt” which includes the two largest cities of Glasgow and Edinburgh. 98% of the Scottish population lives on the mainland and there are some 790 islands, 94 permanently inhabited which are broadly separated into four main island groups: The Outer Hebrides (which make up the Western Isles Health Board), The Inner Hebrides (which are grouped in the Highland Health Board), Orkney and Shetland.

**Table 1:** Health Board demographics, case count, crude and age-standardized incidence rates by Health Board region 2010–2017 ($X_{{13}}^{2} = 94.7$, two-sided *p* < 1 × 10^–13^)

Health Board	Health Board population	Area (km^2^)	Latitude (°N)*	Density (pop/km^2^)	Population F:M ratio	MS case count 2010–2017	Person-years	IR** (95% CI)	IAIR*** (95% CI)	*p* value (Bonferroni-corrected)
Ayrshire and Arran	373,760	3408.95	55.43	109.64	1.09	275	2,990,080	9.20 (8.17–10.35)	9.55 (8.42–10.68)	
Borders	113,880	4742.68	55.60	24.01	1.06	57	911,040	6.26 (4.83–8.11)	6.66 (4.93–8.39)	
Dumfries and Galloway	151,410	6676.05	55.07	22.68	1.06	111	1,211,280	9.16 (7.61–11.04)	9.92 (8.07–11.77)	
Fife	365,300	1373.92	56.12	265.88	1.06	303	2,922,400	10.37 (9.26–11.60)	10.60 (9.41–11.80)	< 0.01
Forth Valley	298,080	2733.66	56.02	109.04	1.06	215	2,384,640	9.02 (7.89–10.31)	9.04 (7.84–10.25)	
Grampian	569,580	8800.94	57.15	64.72	1.02	466	4,556,640	10.23 (9.34–11.20)	10.05 (9.14–10.96)	< 0.01
Greater Glasgow and Clyde	1,135,400	1150.84	55.86	986.59	1.08	670	9,083,200	7.38 (6.84–7.96)	7.21 (6.67–7.76)	< 0.001
Highland	321,660	33,636.70	57.47	9.56	1.04	297	2,573,280	11.54 (10.30–12.93)	12.07 (10.70–13.45)	< 0.001
Lanarkshire	651,620	2246.24	55.49	290.09	1.08	334	5,212,960	6.41 (5.76–7.13)	6.34 (5.66–7.02)	< 0.001
Lothian	836,610	1760.80	55.95	475.13	1.05	478	6,692,880	7.14 (6.53–7.81)	6.85 (6.24–7.47)	< 0.001
Orkney	21,420	1086.21	58.98	19.72	1.02	30	171,360	17.51 (12.24–25.04)	18.35 (11.78 -24.92)	< 0.01
Shetland	23,240	1656.62	60.53	14.03	0.97	20	185,920	10.76 (6.94–16.67)	11.01 (6.18–15.84)	
Tayside	410,250	7684.38	56.46	53.39	1.06	404	3,282,000	12.31 (11.17–13.57)	12.81 (11.56–14.06)	< 0.001
Western Isles	27,690	3268.47	58.21	8.47	1.03	20	221,520	9.03 (5.83–13.99)	9.66 (5.43–13.90)	
Combined						3680				
Scotland^†^	5,299,900				1.06	3716	42,399,200	8.76		

*p* values for individual crude rates determined by comparison of observed rates to distribution of simulated rates under null hypothesis of complete spatial randomness and adjusted for multiple testing. 95% confidence intervals (CIs) calculated based on assumption of events being Poisson distributed. Note, comparison of 95% CIs of indirectly standardized rates between regions—rather than to national rate—is not strictly valid although degree of bias is likely to be small [29]

*Latitude of Health Board administrative centre

**Incidence rates per 100,000 person-years

***Indirect age-standardized (to the national) incidence rate

^†^Including 36 persons excluded from regional calculation due to lack of address

### Regional incidence 2010–2017

**Fig. 2:**
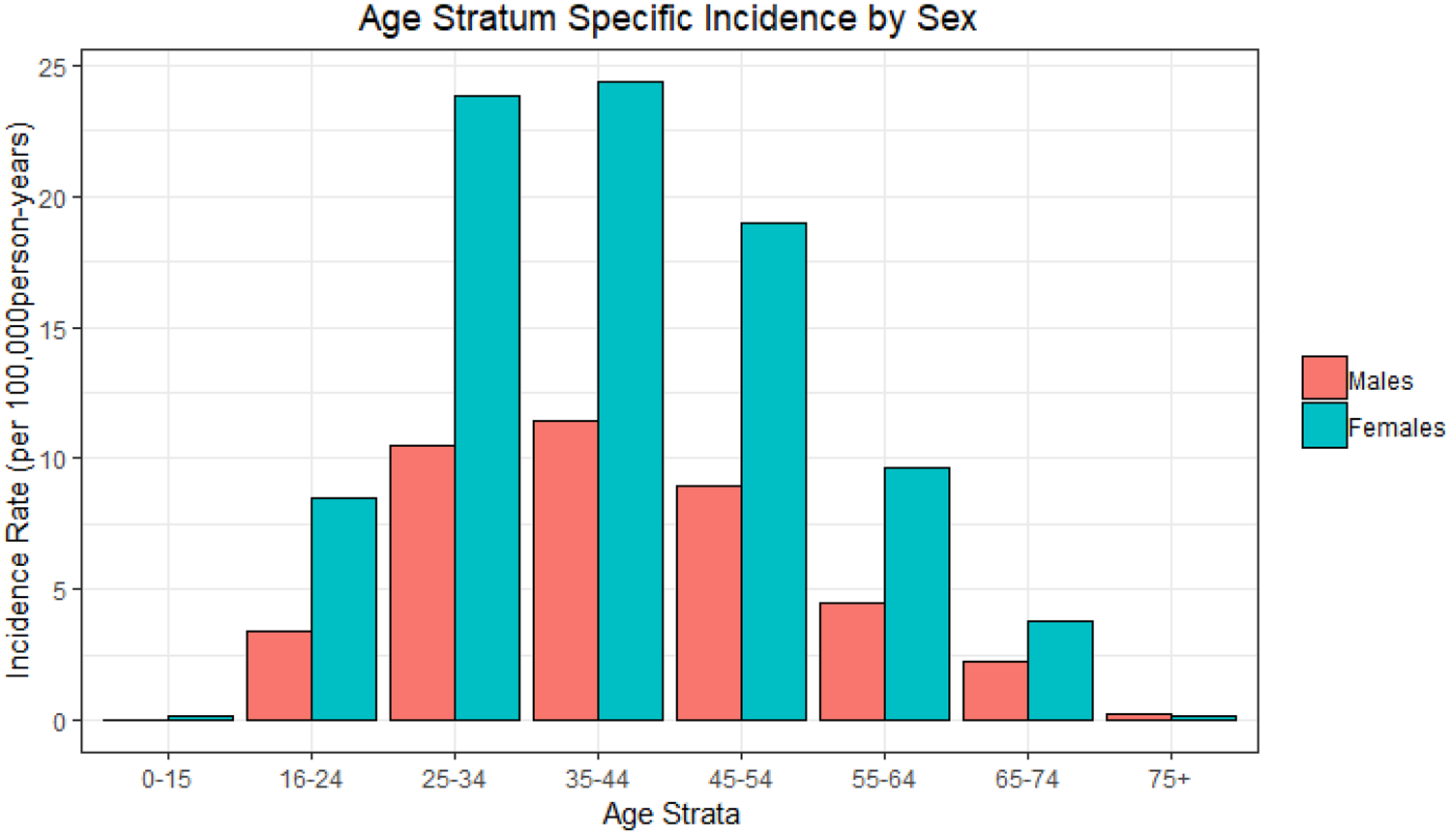
Age- and sex-stratum-specific incidence rates for whole study period 2010–2017

The incidence we refer to throughout this study is the incidence of diagnosis rather than the incidence of disease onset. The crude MS incidence rate for the Scottish population as a whole was 8.76/10^5^ person-years (pys) (directly standardized to ESP, 8.54/10^5^pys). The national crude incidence rate for females was 11.70/10^5^ pys and for males 5.48/10^5^ pys. The ratio of female case count to male was 2.27 (2.14 for adjusted rate ratio). The age range of subjects at the time of diagnosis was 65.8 years with mean age of 41.34 (SD 12.03) years and median of 40 years. Age-specific incidence rates (Fig. 2) were used to calculate indirectly age-standardized incidence rates (supplementary Table 1).

**Fig. 3:**
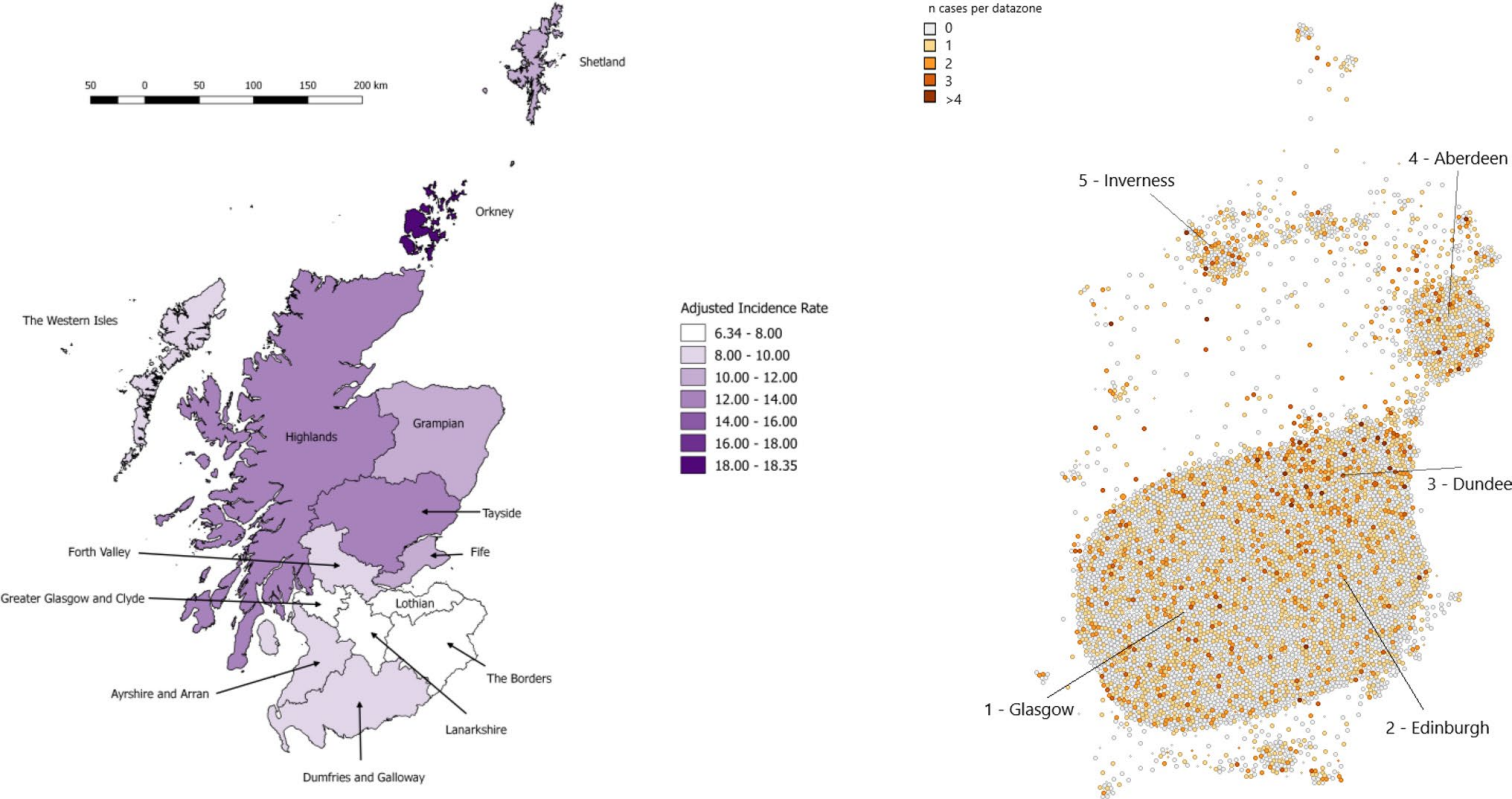
Age-adjusted incidence rates by Health Board for period 2010–2017. (Right) cartogram of count of cases (colour) by population-weighted datazone (size), demonstrating distribution of population-at-risk and cases of MS with concentration in the “central belt” (the region containing Glasgow and Edinburgh). Population-weighted “datazones” are small areal units of geography comprising several postcodes with mean population ~ 800 persons

Crude and indirectly age-standardized rates by Health Board, over the study period, are presented in Table 1 and Fig. 3, demonstrating significant variation in the incidence rate by region ($X_{{13}}^{2} = 94.7$, two-sided *p* < 1 × 10^–13^). Orkney was the highest incidence region in Scotland and there was good agreement between crude and standardized rates indicating that differences in population age-structure were not large contributors to apparent differences in rates. Lanarkshire (6.34), the Borders (6.66), Lothian (6.85) and Greater Glasgow and Clyde (7.21) formed a contiguous band of the lowest incidence regions, with adjusted rates approximately one-third that of Orkney (18.35) and one-half that of Shetland (11.01), Highland (12.07) and Tayside (12.81), the latter being the highest incidence region on the mainland.

Our finding of differences in incidence rates by region agree with those previously estimated by Handel et al. [11] using a different method for case ascertainment [hospital admissions—Scottish Morbidity Records 01 (SMR01)]. We replicate the threefold between-health-board variation described in that study (*r* = 0.85, *p* < 0.001), finding the same regions to be high and low incidence, despite the different patient cohort: 1997–2009 in Handel et al. [11] and 2010–2017 in our study.

**Fig. 4:**
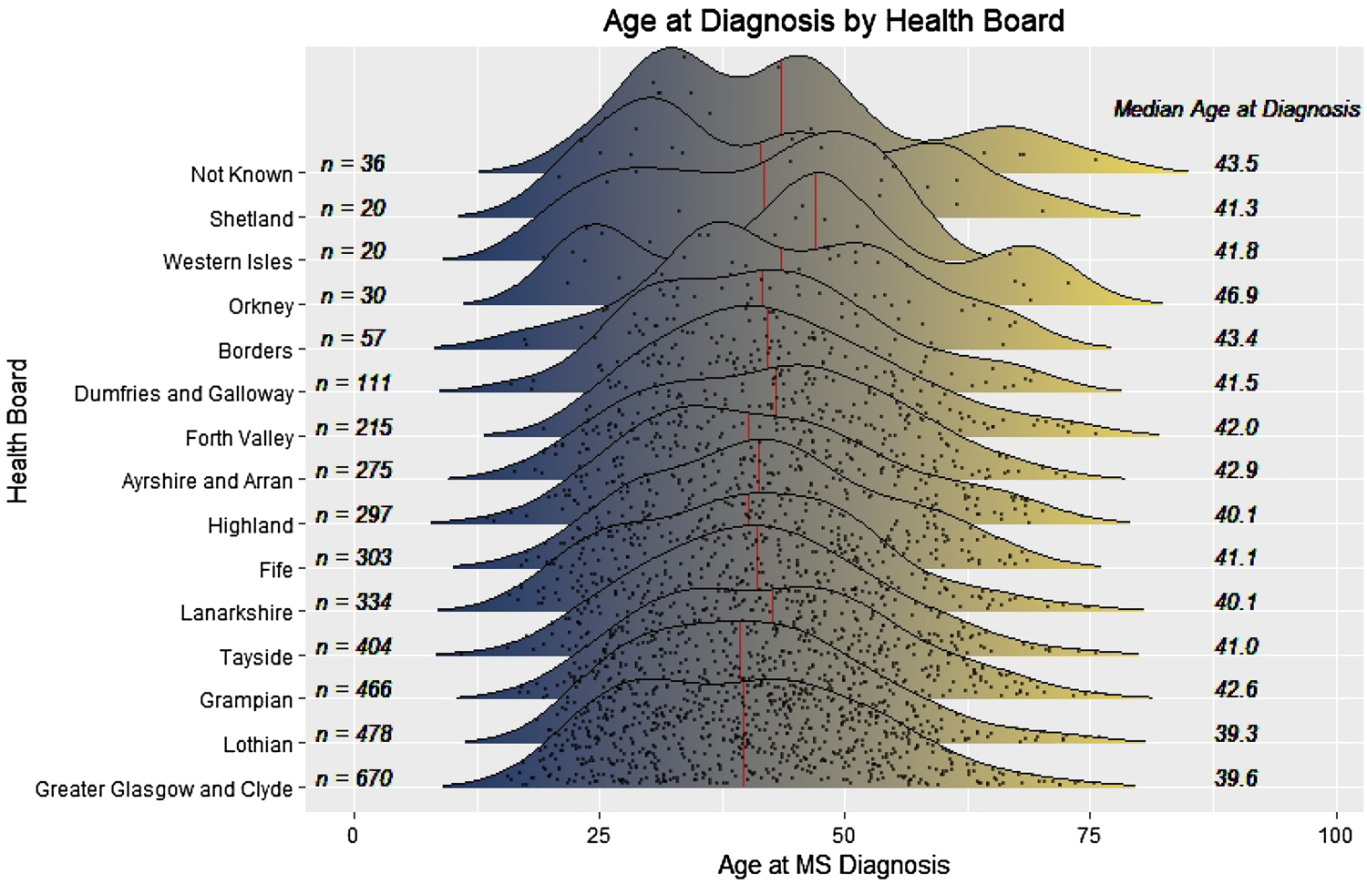
Distribution of age at diagnosis by Health Board region. Points represent cases. Ordered by case number (*n*) over the study period. Vertical red line represents median age at diagnosis for that region

We considered whether ascertainment discrepancies, arising from systematic differences in time from first symptoms to diagnosis, might have contributed to these results and we hypothesized that, if so, this might be reflected in differences in distribution of age at diagnosis (Fig. 4). Whilst visual inspection did not suggest obvious systematic differences between groups, omnibus testing of the age distribution by region using Kruskal–Wallis test demonstrated a significant difference ($X_{{14}}^{2}$ = 33.6, *p* = 0.002). However, this was not unexpected given the large number of cases and power to detect small deviations from uniformity, and in post hoc (Dunn’s) analyses only two (of 91) pairwise comparisons yielded a significant difference after correcting for multiple testing using the false discovery method: the median diagnosis in Glasgow occurred significantly earlier than in Forth Valley (+ 3.3 years, *p* = 0.02) and in Grampian (+ 3.7 years, *p* = 0.01) Health Boards.

In addition to variation in incidence rates, we found variation in the sex ratio of cases between regions (Table 2) albeit this was chiefly driven by two outlier regions in the Borders and Shetland: there was no significant difference across all regions $(X_{{13}}^{2} = 16.6,~p = 0.22)$. The (F:M) sex ratio was most equitable in Shetland 1.0 (*n* = 10:10, lat = 60.39°N) and most inequitable in the borders 4.18 (*n* = 46:11, lat = 55.58°N). Adjusting for differences in regional population age–sex structure accounted for little of this difference. A significant negative linear relationship of sex ratio on latitude was detected by ordinary least squares regression (*β* = − 1.30, *p* = 0.03)—i.e. relatively more females were affected at lower latitudes and the sex ratio was more equitable at higher latitudes. However, again the outliers drive this effect. No significant linear trend with latitude exists amongst the other Health Boards after excluding Shetland and the Borders (*β* = − 0.94, *p* = 0.56) and both areas have low numbers of cases. The effect is similarly not significant when accounting for population in weighted analyses (*β* = − 0.88, *p* = 0.09).

**Table 2:** Crude and age-standardized incidence rates by sex and by Health Board region for whole study period 2010–2017

Health Board	Female					Male					Female:male		
	Count	Person-years	IR*	Standardized-rate** (95% CI)	*p* value (Bonferroni-corrected)	Count	Person-years	IR*	Standardized-rate** (95% CI)	*p* value (Bonferroni-corrected)	Count ratio	Crude rate ratio	IAIR Ratio**
Ayrshire and Arran	197	1,558,920	12.64	13.03 (11.20–14.85)		78	1,431,160	5.45	5.70 (4.44–6.97)		2.53	2.32	2.28
Borders	46	469,480	9.80	10.44 (7.42–13.46)		11	441,560	2.49	2.64 (1.08–4.21)	< 0.05	4.18	3.93	3.95
Dumfries and Galloway	77	624,248	12.33	13.29 (10.32–16.26)		34	587,032	5.79	6.28 (4.17–8.40)		2.26	2.13	2.12
Fife	212	1,506,600	14.07	14.37 (12.44–16.31)	< 0.05	91	1,415,800	6.43	6.57 (5.22–7.93)		2.33	2.19	2.19
Forth Valley	156	1,226,720	12.72	12.72 (10.72–14.72)		59	1,157,920	5.10	5.12 (3.82–6.43)		2.64	2.50	2.48
Grampian	308	2,302,464	13.38	13.18 (11.71 0 14.66)	< 0.05	158	2,254,176	7.01	6.87 (5.80–7.95)	< 0.05	1.95	1.91	1.92
Greater Glasgow and Clyde	478	4,717,888	10.13	9.92 (9.03–10.81)	< 0.001	192	4,365,312	4.40	4.30 (3.69–4.91)	< 0.01	2.49	2.30	2.31
Highland	210	1,312,416	16.00	16.70 (14.43–18.96)	< 0.00001	87	1,260,864	6.90	7.23 (5.71 - 8.75)	< 0.05	2.41	2.32	2.31
Lanarkshire	236	2,699,432	8.74	8.63 (7.52–9.73)	< 0.000001	98	2,513,528	3.90	3.87 (3.11–4.64)	< 0.01	2.41	2.24	2.23
Lothian	326	3,435,376	9.49	9.12 (8.13–10.11)	< 0.00001	152	3,257,504	4.67	4.47 (3.76–5.19)	< 0.05	2.14	2.03	2.04
Orkney	21	86,480	24.28	25.34 (14.49–36.18)	< 0.05	9	84,880	10.60	11.14 (3.86–18.42)		2.33	2.29	2.27
Shetland	10	91,616	10.92	11.21 (4.25–18.16)		10	94,304	10.60	10.82 (4.11–17.53)		1.00	1.03	1.04
Tayside	263	1,692,856	15.54	16.20 (14.24–18.16)	< 0.00001	141	1,589,144	8.87	9.21 (7.69–10.74)	< 0.000001	1.87	1.75	1.76
Western Isles	14	112,304	12.47	13.61 (6.47–20.75)		6	109,216	5.49	5.75 (1.15–10.36)		2.33	2.27	2.37
Scotland	2554	21,836,800	11.70			1126	20,562,400	5.48			2.27	2.14	2.23

*p* values represent Bonferroni-corrected probabilities of observed rates having arisen under a null hypothesis of spatial randomness of the distribution of cases across Scotland

*Incidence rates per 100,000 person-years

**Indirect age-standardized incidence rate (IAIR) (95% confidence intervals)

We found that the lifetime risk of MS in Scotland is 6.55 per 1000 persons for the country as a whole and, whilst the sex ratio varies regionally, in each region the lifetime risk for females is higher than for males. However, the degree of variation in incidence is such that the lifetime risk for males in Shetland (8.63), Orkney (7.94) and Tayside (6.89) is higher than for females in some other areas (for example, Lanarkshire (6.60), Lothian (6.98), and Greater Glasgow and Clyde (7.56)). Table 3 presents the calculated male, female and combined lifetime risk (CIR) by Health Board.

**Table 3:** Cumulative incidence rates (approximation of hypothetical lifetime risk)

Health Board	CIR (per/1000)		
	All	Female	Male
Ayrshire and Arran	7.24	9.97	4.28
Borders	4.98	8.12	1.65
Dumfries and Galloway	7.74	10.41	4.90
Fife	7.97	10.89	4.90
Forth Valley	6.82	9.70	3.73
Grampian	7.58	9.98	5.17
Greater Glasgow and Clyde	5.45	7.56	3.22
Highland	9.36	13.05	5.60
Lanarkshire	4.80	6.60	2.91
Lothian	5.17	6.98	3.30
Orkney	13.86	19.88	7.94
Shetland	8.66	8.68	8.63
Tayside	9.61	12.22	6.89
Western Isles	7.51	10.80	4.38
National	6.55	8.90	4.10

## Discussion

This work provides the first report of standardized regional incidence rates for MS in Scotland based on the recently established SMSR which is the result of substantial far-sighted public investment. The high level of completeness of these data (99% for age, sex and address at diagnosis) is a result of mandatory reporting, the CHI system which incorporates sex and date of birth, and the importance of current address for the functioning of normal service within the NHS.

This study builds on a strong track record of several decades of MS epidemiological surveys in Scotland and utilizes the SMSR for its particular advantages for aetiological investigation: chiefly, that the register is truly national, is aiming for complete ascertainment, and has a prospective, incidence design. Until the recent emergence of disease-modifying treatments (DMTs), prevalence, mortality, hospital admission and even disability pensioning rates were comparable across regions relatively unaffected by differences in medical services available. However, an improved therapeutic arsenal now potentially confounds comparisons of these rates as regional discrepancies in care provision or practice may now also affect disability, admissions and survival. A strength, therefore, is that the SMSR captures incidence data, which is inherently more biologically meaningful than other rates—especially in the era of DMTs.

The national crude incidence rate of 8.71/10^5^ pys confirms Scotland as a high-incidence nation, although not the highest reported internationally. The standardized incidence we report (8.54) is lower than recent studies from other high-income countries. For example, in Denmark at 9.43 (95% CI 9.17–9.69) [16] and Wales at 9.10 (95% CI 8.80–9.40) [17] per 10^5^ pys. However, given the incidence register excludes possible MS and all cases are confirmed as definite by the diagnosing neurologist, it is likely that the rates we present reflect a lower bound on the estimate of the full burden of the condition in Scotland. Further, newly recognized clinical entities (such as neuromyelitis optica spectrum disorders), which in previous generations may have been included for calculation of MS rates, will now not be included. Caution may be merited in comparing these rates to those obtained in other settings by different methods. Our data support the assessment of the Scottish Public Health Observatory, that the SMSR is now the gold standard estimates of incidence in Scotland [18].

Given substantial regional variation, previous published estimates extrapolating prevalence from samples taken from single regions and/or using other methods may have erroneously estimated the burden of the disease in Scotland. Choice of index region and method of ascertainment are important potential sources for bias. A previous estimate, for example, of 10,000 prevalent cases would only be plausible if our figures represented an epidemic since 2010 and we suspect this to be a large underestimate [19]. Another estimate of 13,328 pwMS in 2010 derived from GP data supported by hospital episode data [10] appears more plausible. However, by roughly extrapolating prevalence from these incidence data, using life-expectancy and estimates of effects of MS diagnosis on early mortality [20], we suspect that this may also fall short. A recent study, using administrative data in the USA, has suggested that the prevalence of MS there may be as high as double that previously reported [21]. Similarly, we suspect that the burden of MS in Scotland may have been underestimated.

Our finding of differences in incidence rates by region agree with those estimated by Handel et al. [11] using different methods (hospital admissions) and our simulations demonstrate these differences are unlikely to have arisen due to chance (Table 1 and supplementary Fig. 1). This correlation between studies across distinct periods is externally consistent with reports of regional variability persisting across generations in other countries (where intergenerational correlation has been reported as *r* ~ 0.8) [6]. The consistency across two methods—our study corroborating their findings of threefold variation (*r* = 0.85, *p* < 0.001), finding the same regions to be high and low incidence, whilst using a more robust dataset, and in a different patient cohort—leads us to conclude that regional variation in Scotland is real, persistent and not explained by chance or bias.

In fact, a limitation of our methods is that address at the time of diagnosis is presumed to be an imperfect proxy for address at the time of onset and at the time of exposure to environmental risk factors [22]. Our study would thus be strengthened by data on pre-diagnosis location of residence. We would expect the imperfect correlation between location of residence at diagnosis and exposure to have diluted regional variation and so, despite finding large variations, we may have underestimated the extent of this effect.

The regional variation, even on the relatively ancestrally homogenous mainland, we find to be surprisingly large. Regional differences in CIR, which can be interpreted as a hypothetical lifetime risk, range substantially, from 19.9/1000 for females in Orkney (55.98°N) to 1.7/1000 for males in the Borders (55.60°N) (Table 3). And in 6 of 14 Scottish Health Boards the lifetime risk for women exceeds 1%. However, surprisingly, variation in incidence and sex ratio is of the degree that in some Health Boards of Scotland the lifetime risk for men exceeds the lifetime risk for women in other regions, despite an overall sex ratio of 2.3 (at the expense of women).

The variation in sex ratio between regions may be the result of chance. The negative correlation between sex ratio and latitude (greater proportion of females at low latitudes) in our unweighted analyses is in line with international trends [23]. However, the Borders and Shetland are outlier regions. No convincing linear relationship between sex ratio and latitude exists outwith these areas or on weighted analyses. However, the sex ratio of MS has been reported to have changed over decades, for example, in Canada [24] and Denmark [16], implicating changes in an environmental risk factor(s) for which males and females have differential sensitivity or exposures. Therefore, analysis of the regional sex ratio of future cases may be informative in demonstrating that these outliers arose due to chance, as we cannot yet exclude the possibility that they are the product of some local environmental factor.

Latitude predicts only a small proportion of the total variability in Scotland, but does remain significant even after controlling for population age–sex structure. This is perhaps in contrast to other Western European countries where it has been suggested that latitude’s association with incidence has diminished over time or was artefactually overestimated due to ascertainment issues or failure to standardize populations [25]. However, as northern populations in Scotland are typically more rural and have more outdoor work, it has been questioned—with empirical support—as to whether vitamin D levels robustly correlate with latitude in Scotland [26]. In addition, the lower incidence in Shetland, by far the most northerly point in Scotland, compared to Orkney and Tayside, raises further questions [26]. The remoteness of Shetland, with its distinct ancestry and—to an extent—culture, could explain the deviation from the general trend. However, this phenomenon, of a reversal of the latitude effect at extreme high latitudes, is actually consistent with international reports. For example, the same reversal has been noted in Scandinavia and Russia [25]. Nevertheless, even if some other factor explains the proportion of variation associated with latitude in the rest of Scotland, it is our opinion that this would not be any evidence against the importance of hypovitaminosis D in general, given international trends and multiple lines of evidence [27]. Hypovitaminosis D is widely prevalent in Scotland and may significantly contribute to the burden of MS [26].

This study uses routinely collected data with attendant methodological limitations. For example, it is not possible to exclude some regional variation in case ascertainment, although overall ascertainment is thought to be high suggesting that any such variation is probably small. Also, whilst the mean age at diagnosis (41.34 years) was somewhat higher in our study than in a clinically validated cohort from the UK MS register that does not include pwMS in Scotland (37.4 years) [28], the similar patterns of age at diagnosis across Health Boards is an argument against there being systematic differences in diagnostic efficiency by region in Scotland. Possible explanations for this discrepancy include: differences in proportions of patients by type of MS at onset in these two cohorts, an ageing population, truncation of the SMSR at young ages due to exclusion of cases with paediatric onset (age < 16 years old), and/or delays in diagnosis due to waiting times to see a neurologist. For example, it may be that younger median age at diagnosis in Glasgow, the only region where post hoc testing identified a significant difference (relative to two other areas), could, in part, reflect access to tertiary neurological care or waiting times to see neurologists. However, local differences in services and diagnostic preferences (e.g. propensity to request a lumbar puncture prior to diagnosis), and/or net rural-to-urban migration skewing the population-at-risk, will also be contributory factors, in addition to chance variation.

## Conclusion

This paper presents the first 8 years of the SMSR, a prospective national incidence register. We confirm the high incidence of MS in Scotland, suggest that previous estimates of prevalence are likely underestimates, and corroborate previous reports of threefold variation by Health Board region suggesting these differences are both persistent and real. This has implications for service provision and supports further study to better understand the basis of regional variability. It raises the possibility that biologically important risk factors may be variably distributed in Scotland, at least regionally, and future work is now possible to determine if variation exists at a more local scale.

## Electronic supplementary material

Below is the link to the electronic supplementary material.

Supplementary file1 (DOCX 69 kb)
