# Supplementary material for: Regional variation in the incidence rate and sex ratio of multiple sclerosis in Scotland 2010–2017: findings from the Scottish Multiple Sclerosis Register
Source: J Neurol. 2019 Jun 11;266(10):2376–86. doi: 10.1007/s00415-019-09413-x (PMC6765473; doi:10.1007/s00415-019-09413-x)

**Supplementary Table 1.** Age- and sex- stratum specific incidence rates by Health Board region for whole study period 2010-2017.

|  | Male |  |  |  | Female |  |  |  |
| --- | --- | --- | --- | --- | --- | --- | --- | --- |
| Age Stratum | cases | n^**^ | person-years | rate | cases | n^**^ | person-years | rate |
| 0-15 | * | * | * | 0.03 | * | * | * | 0.14 |
| 16-24 | 86 | 316699 | 2533592 | 3.39 | 213 | 314361 | 2514888 | 8.47 |
| 25-34 | 277 | 330036 | 2640288 | 10.49 | 648 | 340099 | 2720792 | 23.82 |
| 35-44 | 325 | 355342 | 2842736 | 11.43 | 730 | 374423 | 2995384 | 24.37 |
| 45-54 | 275 | 384905 | 3079240 | 8.93 | 612 | 403181 | 3225448 | 18.97 |
| 55-64 | 118 | 327388 | 2619104 | 4.51 | 263 | 340999 | 2727992 | 9.64 |
| 65-74 | 41 | 227079 | 1816632 | 2.26 | 78 | 257701 | 2061608 | 3.78 |
| 75+ | * | * | * | 0.23 | * | * | * | 0.15 |
| Crude Incidence |  |  |  | 5.47 |  |  |  | 11.68 |

^*^ Count suppressed/redacted as per ISD policy on potentially identifiable low-count data

^**^ n = number of population at risk

**Supplementary Table 2:** Age groups and standard populations

| Scottish Government (Scottish Index of Multiple Deprivation) age categories, here used for indirect standardization and CIR. | | | 19 Age Group (5 year) – European Standard Population 2013 | | | 18 Age Group (5 year) -- European Standard Pop (1976) | | |
| --- | --- | --- | --- | --- | --- | --- | --- | --- |
| Age Group | Ages (y) | Weigth (wi) for CIR calculation | Age Group | Ages (y) | Pop | Age Group | Ages (y) | Pop |
| 1 | 0-15 | 15 | 1 | 0-4 | 5000 | 1 | 0-4 | 8000 |
| 2 | 16-24 | 10 | 2 | 5-9 | 5500 | 2 | 5-9 | 7000 |
| 3 | 25-34 | 10 | 3 | 10-14 | 5500 | 3 | 10-14 | 7000 |
| 4 | 35-44 | 10 | 4 | 15-19 | 5500 | 4 | 15-19 | 7000 |
| 5 | 45-54 | 10 | 5 | 20-24 | 6000 | 5 | 20-24 | 7000 |
| 6 | 55-64 | 10 | 6 | 25-29 | 6000 | 6 | 25-29 | 7000 |
| 7 | 65-74 | 10 | 7 | 30-34 | 6500 | 7 | 30-34 | 7000 |
| 8 | 75+ | (see below) | 8 | 35-39 | 7000 | 8 | 35-39 | 7000 |
|  |  |  | 9 | 40-44 | 7000 | 9 | 40-44 | 7000 |
| Scottish life expectancy at birth (y) | | Weight for Age Group 8 CIR calc | 10 | 45-49 | 7000 | 10 | 45-49 | 7000 |
| Females | 81.2 | 7.2 | 11 | 50-54 | 7000 | 11 | 50-54 | 7000 |
| Males | 77.1 | 3.1 | 12 | 55-59 | 6500 | 12 | 55-59 | 6000 |
| Combined | 79.5 | 5.15 | 13 | 60-64 | 6000 | 13 | 60-64 | 5000 |
|  |  |  | 14 | 65-69 | 5500 | 14 | 65-69 | 4000 |
|  |  |  | 15 | 70-74 | 5000 | 15 | 70-74 | 3000 |
|  |  |  | 16 | 75-79 | 4000 | 16 | 75-79 | 2000 |
|  |  |  | 17 | 80-84 | 2500 | 17 | 80-84 | 1000 |
|  |  |  | 18 | 85-89 | 1500 | 18 | 85+ | 1000 |
|  |  |  | 19 | 90+ | 1000 |  |  |  |

**Supplementary Figure 1.** Crude incidence rates 2010-2017 against rates of hospital admissions in Handel et al 2011[12]. Pearson correlation coefficient: r = 0.85, p<0.001. Trendline fitted with ordinary least squares regression, and shaded area represents 95% CI.


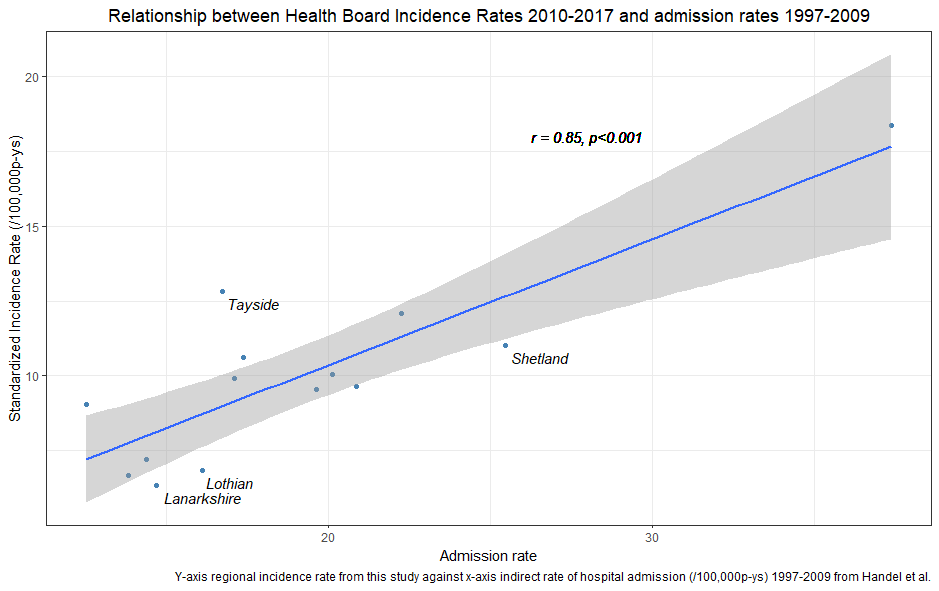

Supplement: Supplementary file 1 — Supplementary file1 (DOCX 69 kb) [file 415_2019_9413_MOESM1_ESM.docx]
